# Is there an association between left atrial outpouching structures and recurrence of atrial fibrillation after catheter ablation?

**DOI:** 10.1371/journal.pone.0276369

**Published:** 2022-10-27

**Authors:** Erkan Celik, Nils Große Hokamp, Lukas Goertz, Wolfgang Fehske, Dinh Quang Nguyen, Lutz Lichtenberg, Robert Peter Reimer, David Maintz, Christoph Düber, Tobias Achenbach

**Affiliations:** 1 Department of Diagnostical and Interventional Radiology, St. Vinzenz Hospital, Cologne, Germany; 2 Department of Diagnostic and Interventional Radiology, University of Cologne, Faculty of Medicine and University Hospital Cologne, Cologne, Germany; 3 Department of Internal Medicine III Cardiology, St. Vinzenz Hospital, Cologne, Germany; 4 Department of Diagnostical and Interventional Radiology, Johannes Gutenberg-University Medical Centre, Mainz, Germany; 5 Department of Diagnostic and Interventional Radiology, Lahn-Dill-Kliniken, Wetzlar, Germany; University of Minnesota, UNITED STATES

## Abstract

**Objective:**

The aim of this study was to investigate the impact of left atrial diverticula (LADs), left sided septal pouches (LSSPs) and middle right pulmonary veins (MRPVs) on recurrent atrial fibrillation (rAF) in patients undergoing laser pulmonary vein isolation procedure (PVI).

**Material and methods:**

This retrospective study enrolled 139 patients with pre-procedural multiple detector computed tomography (MDCT) imaging and 12 months follow-up examination. LADs, LSSPs and MRPV were identified by two radiologists on a dedicated workstation using multiplanar reconstructions and volume rendering technique. Univariate and bivariate regression analyses with patient demographics and cardiovascular risk factors as covariates were performed to reveal independent factors associated with rAF.

**Results:**

LADs were recorded in 41 patients (29%), LSSPs in 20 (14%) and MRPVs in 15 (11%). The right anterosuperior wall of the left atrium was the most prevalent location of LADs (68%). rAF occured in 20 patients, thereof, 15 exhibited an outpouching structure of the left atrium (LAD: 9, LSSP: 2 and MRPV: 3). Presence of an LAD (HR: 2.7, 95%CI: 1.0–8.4, p = 0.04) and permanent AF (HR: 4.8, 95%CI: 1.5–16.3, p = 0.01) were independently associated with rAF.

**Conclusions:**

LAD, LSSP and MRPV were common findings on pre-procedural cardiac computed tomography. LADs were revealed as potential independent risk factor of rAF, which might be considered for treatment planning and post-treatment observation.

## 1. Introduction

Atrial fibrillation (AF) affects 1% to 2% of the general population, thus representing the most prevalent cardiac arrhythmia [1–3]. The lifetime risk of developing AF is >30% for both men and women [4]. The inefficient contraction of the left atrium creates a turbulent blood flow that increases the risk of developing cardiac thrombosis. AF is associated with a nearly 5-fold increased risk for ischemic stroke [5, 6]. The main approaches to the treatment of AF are rate control, rhythm control and stroke prevention [3, 7]. AF occurs due to complex electrical defects in the atrium including a rapidly firing focus, complex multiple reentrant circuit or rotors [8]. The development of these electrical defects is boosted by changes of three categories: electrical remodeling, structural remodeling and anatomic remodeling. As pulmonary vein sleeves are the most frequent anatomical source of ectopic cardiac pacemaker cells with proarrhythmogenic potential, catheter ablation is frequently used to uncouple these cells [9–11]. Ablational therapies not only include the PV but other ablation sites such as the left atrial roof, the posterior wall, the interatrial septum or the mitral isthmus line [12]. To improve the success rates and to avoid complications during ablation, knowledge on outpouchings of the left atrium and pulmonary veins is desirable for procedural planning, all of which can be reliably visualized by multiple detector computed tomography (MDCT), [13–15]. Middle right pulmonary veins (MRPV) are described as one of the most common variations of the PV, being present in almost 20% of patients and baring the risk to initiate AF [16, 17]. Left atrial diverticula (LAD) are defined as pouchy evaginations of the left atrial cavity with a broad body, wide neck and smooth contour [18]. LAD are a common finding in cardiac-CT, being present in up to 36% of patients undergoing AF ablation. They are reported to serve as an extra cardiac pacemaker focus, and comprise a risk factor for intracavitary thrombosis and cardiac perforation during ablation [19–23]. According to histopathological analyses, LADs contain trabeculated myocardium with the same wall structure as the surrounding myocardium and were firstly reported to show ectopic activity in a documented case report in 2009 [24, 25]. Left sided septal pouches (LSSP) are considered structures that occur when the patent foramen ovale (PFO) is absent but the septum primum and septum secundum are not completely fused [26]. Their clinical role is unclear however some data indicate that LSSP could be a trigger for atrial fibrillation and cryptogenic stroke [26–29].

The aim of this study is to investigate if there is a correlation between LAD, MRPV, LSSP and recurrence of atrial fibrillation (rAF) following pulmonary vein isolation.

## 2. Material and methods

The Ethics Commission of Cologne University’s Faculty of Medicine approved this retrospective, single-center study (reference number 19–1439). Due to the retrospective nature of the study, a written informed consent was waived.

### 2.1 Patient population

A structured database query of the picture archiving and communication system (PACS) revealed all patients who received a contrast enhanced electrocardiogram-gated multidetector computed tomography of the heart between January 2013 to September 2014 prior to pulmonary vein isolation. All ablations were performed by laser pulmonary vein ablation technique. This resulted in 150 patients screened for study inclusion. Patients with incomplete imaging (N = 6), incomplete ablation procedure with incomplete circumferential lines around the pulmonary veins during catheter ablation (N = 3) or incomplete follow-up visits (N = 5) were excluded from further analysis. In all three cases of incomplete ablation procedure, the anatomy of the esophagus was unfavorable and a complete ablation could not be performed to avoid heat damage.

### 2.2 CT techniques

Cardiac computed tomography was performed using a dedicated scan-protocol with visual triggering of the contrast medium bolus in the left atrium. All studies were carried out using a 64 row MDCT scanner (Discovery CT750 HD, GE Healthcare, Waukesha, WI). Scanning was performed in craniocaudal direction from above the aortic arch to the diaphragm using prospective ECG-gating. Using a power injector, 60 ml of an intravenous contrast agent (Imeron 400 mg/mL, Bracco, Milan, Italy) was administered at a flow rate of 5.0 ml/s through a cubital vein. The contrast bolus was followed by a saline chaser of 50ml at the same flow. No premedication was given. Further scan parameters comprised: 120kV, 200 mAs with tube current modulation. Images were reconstructed using the following parameters: CardIQ Xpress (ImageWorks^™^, GE Healthcare, Waukesha, WI), slice thickness 0.625 mm, section increment 0.1 mm.

### 2.3 CT data post processing and image analysis

**Fig 1:**
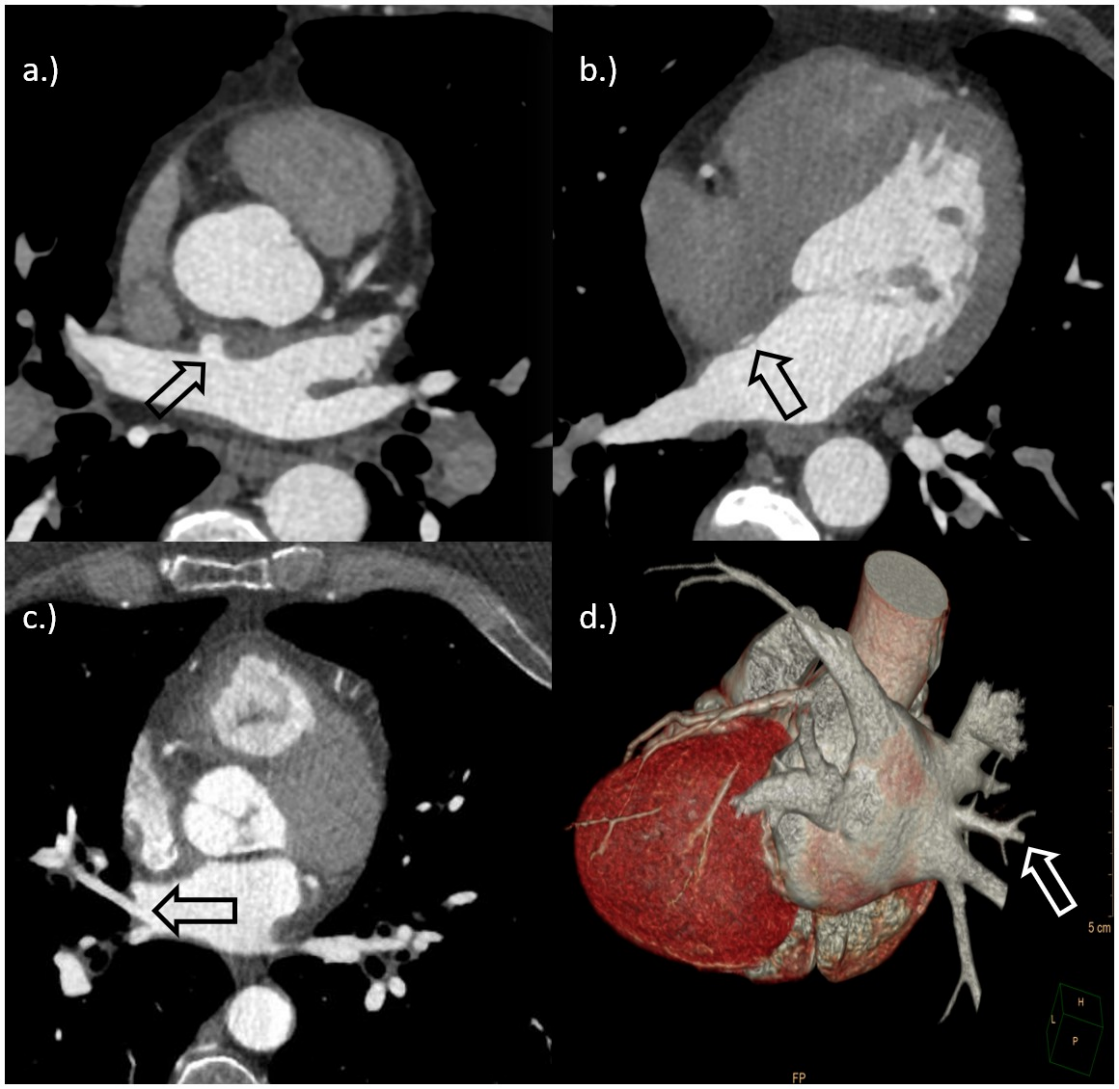
a.) Cardiac-CT of a 55 years old female patient with AF. The arrow shows a single left atrial diverticulum in the anterosuperior wall. b.) Cardiac-CT of a 54 years old female patient with AF. The arrow shows a left-sided septal pouch. c.) + d.) Cardiac-CT and three-dimensional volume rendering of the heart of a 44 years old male patient with AF. The arrows (white and black) show an accessory pulmonary vein merging into the left atrium from the right.

All images were transferred to a dedicated cardiac work station (GE Medical, Advanced Workstation 4.6 and Cardiac iQ Express, GE Medical). Two radiologists with 5 and 15 years of experience in cardiac imaging (EC, TA) reviewed the images in consensus using multiplanar reconstructions and volume-rendering techniques. Radiologists were blinded to all clinical data except age and gender. Presence of LAD including the location, LSSP and MRPV were recorded (Fig 1). Left atrial diverticula were defined as pouchy evaginations of the left atrial cavity with a broad body, wide neck and smooth contour [18].

### 2.4 Ablation procedure

Pulmonary vein isolation was performed with the “CardioFocus HeartlightTM” system using laser energy (Heartlight^™^, CardioFocus, Marlborough, MA, US). It is a visually controlled laser ablation system consisting of a console, a 15-Charrière outer diameter steerable transseptal sheath, a reusable 2-Charrière endoscope, and the 12-Charrière catheter containing a size-adjustable balloon at the distal end. The console is used to deliver the laser energy, visualize the endoscopic image, and control the balloon size [30]. Balloon diameters can be adjusted between 9 mm and 35 mm. Ablation is induced by a 980 nm diode laser that generates a 30° arc of energy.

All procedures were carried out under anesthesiologic supervision in analgesia with propofol and remifentanil in a cardiac catheter laboratory with a biplane, swiveling X-ray fluoroscopy unit. During the procedure, the patients were heparinized in a controlled manner over the activated clotting time with a target value between 300 and 350 seconds to avoid thromboembolic complications. The measurements were made every 10 to 20 minutes. The left atrial appendage was checked for thrombus using transesophageal echocardiography. Access was via the femoral vein on both sides. A four-pole diagnostic catheter was placed in the right ventricle and a six-pole diagnostic catheter in the coronary sinus after puncturing the left femoral vein and using two 7 F-sheaths. Access to the left atrium was made after puncturing the right femoral vein and placing a long transseptal sheath (SL-0 ^™^, St. Jude Medical, St. Paul, USA) and subsequent fluoroscopic and transesophageal echocardiographically controlled single puncture of the atrial septum using a transeptal needle (BRK) XS 71cm ^™^, St. Jude Medical, St. Paul, USA). This was followed by a semi-selective angiographic display of all pulmonary veins in left anterior oblique 50° (LAO) and right anterior oblique 25° (RAO) with high-frequency stimulation of the right ventricle (rapid pacing), with a cycle length of between 240 ms and 300 ms. An esophageal temperature probe (SensiThermTM, St. Jude Medical, St. Paul, USA) was used to measure the esophageal temperature in all patients. To avoid phrenic lesions, the compound motor action potential method (CMAP) was used to isolate the right pulmonary veins. If present, MRPV were targeted by laser balloon. Accessory PVs were not specifically targeted, because they were not present. During the ablation of the right pulmonary veins, the phrenic nerve was stimulated with an electrophysiological diagnostic catheter (Response TM, St. Jude Medical, St. Paul, USA) in the superior vena cava and the resulting diaphragmatic electromyogram monitored with surface electrodes. After ablation is complete, isolation success is verified using a multipolar circular mapping catheter (LASSOTM, Johnson & Johnson, New Brunswick, USA).

### 2.5 Clinical information and patient follow-up

The following clinical parameters at time point of procedure were collected by a medical chart review of the hospital information system (ORBIS; Agfa HealthCare, Bonn, Germany): age, gender, presence of diabetes mellitus, periphery artery disease, atrial fibrillation, heart failure, hypertension and hyperlipidemia. Additionally, left atrial diameters were given in patients records after transthoracic echocardiogram. The antiarrhythmic drug regimen was as follows: If the patient presented with an existing antiarrhythmic medication (Amiodarone, Betablockers, single- or multi-channel blocker) prior to the intervention, it was continued for three additional months after PVI (blanking period). If the patient was not taking any antiarrhythmic medication, no antiarrhythmic medication was started after PVI. Clinical follow-up visits were scheduled 3, 6 and 12 months after ablation and included a holter monitoring for seven days. AF recurrence was defined as the presence of any episode of atrial tachycardia or AF lasting 30 s or more on 12-lead electrocardiogram. Patients with incomplete follow-up visits were excluded from further analysis (n = 5).

### 2.6 Statistical analysis

Categorical variables are presented as numbers with percentages and compared using the Chi-square or the Fisher exact test, when appropriate. Continuous variables are reported as mean ± standard deviation and compared using the two-sided Student’s t-test and the Mann-Whitney-U test, when appropriate. Cut-off values were evaluated with receiver-operating-characteristics curve analysis by determination of the Youden index. Factors with a p-value < 0.2 in the univariate analyses were entered into a binary logistic regression to identify independent factors associated with the respective outcome measure. Statistical significance was defined as p < 0.05. All analyses were performed using dedicated software (JMP v14, SAS Institute, Cary, NC, USA and SPSS Statistics v25, IBM, Armonk, NY, USA).

## 3. Results

The final study cohort consisted of 139 patients that received 139 cardiac ablations due to atrial fibrillation (median age 64.4 ± 12.7 years, 88 men). The most prevalent cardiovascular risk factors were coronary artery disease and arterial hypertension (63% each). The mean LAEDD was 4.4 ± 0.4 cm.

### 3.1 Prevalence of outpouching structures

**Fig 2:**
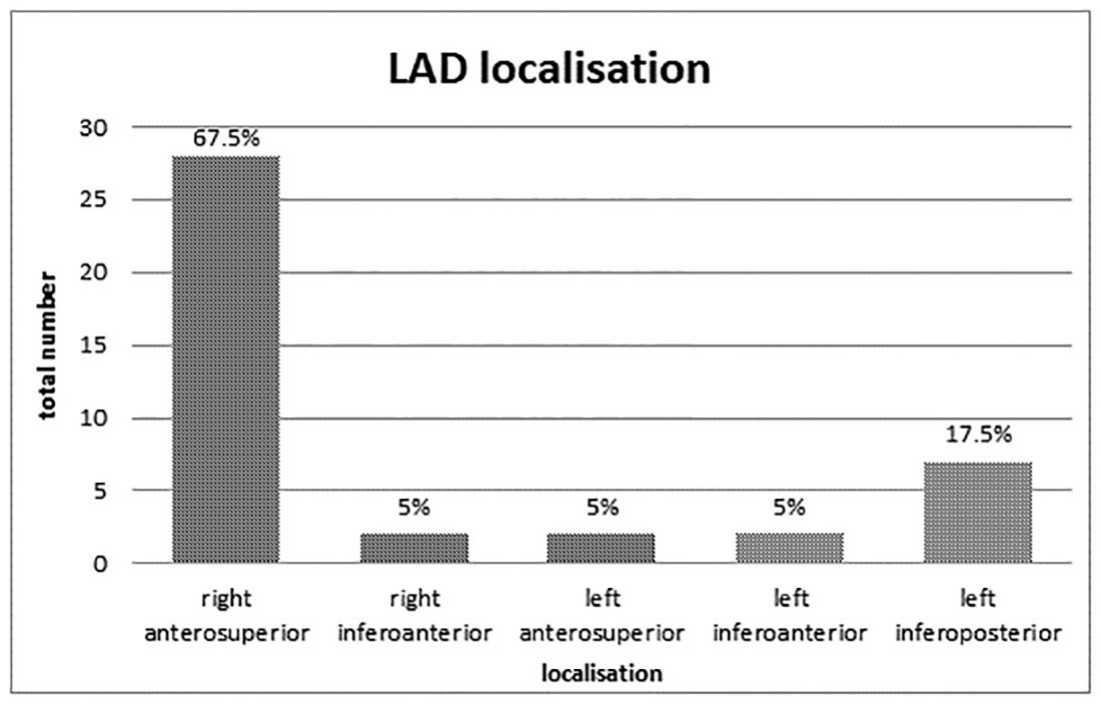
Overview of the total amount of left atrial diverticula and their localisation.

A total of 41 (29%) LADs were identified (28 men; 12 women). Multiple LADs in one patient were not found. In 28 of these cases (68%), the LAD was located in the right anterosuperior wall of the left atrium, 7 (18%) in the left inferoposterior wall, and each 2 (5%) in the right inferoanterior, the left anterosuperior and the left inferoanterior wall, respectively (Fig 2).

Most of the LAD were located close to the ostia of the pulmonary veins.

Accessory pulmonary veins were not found in the present study cohort. MRPVs were found in 15 (11%) cases and LSSPs in 20 (14%).

### 3.2 Correlation with recurrent atrial fibrillation

The overall rAF rate was 14% (20/139), including 8 PV reconnections and unclear cause in 12. Of these 20 patients, 13 (65%) had an outpouching of the left atrial anatomy: Nine (45%) had an LAD, 3 (11%) an MRPV and one (5%) an LSSP. In the univariate analysis, the presence of an LAD was by trend associated with rAF (HR = 2.2, 95%CI = 0.8–5.9; p = 0.1). The other two outpouching types were not significantly associated with rAF (Table 1). Among patient demographics and cardiovascular risk factors, persistent AF (HR = 2.8, 95%CI = 1.0–8.0; p = 0.043) and left atrial diameter > 4.6 cm (HR = 3.5, 95%CI = 1.2–10.2; p = 0.017) were further factors associated with rAF (Table 1). Covariates with a p-value < 0.2 in the univariate analysis were entered into the bivariate logistic regression model. Hereby, persistent AF and presence of a LAD were confirmed as independent risk factors for rAF (Table 2).

**Table 1:** Patient characteristics with and without AF recurrence.

Parameter	AF recurrence (n = 20)	No recurrence (n = 119)	P-value
Age (years; mean ± SD)	62.6 ± 14.1	64.7 ± 12.5	0.60
Sex (*male* vs. female), n (%)	*12 (60%)*	*76 (64%)*	0.80
AF subtype (*persistent* vs. paroxysmal), n (%)	*7 (35%)*	*19 (16%)*	**0.04** *
LAD, n (%)	9 (45%)	32 (27%)	0.10*
LSSP, n (%)	1 (5%)	19 (16%)	0.20
MRPV, n (%)	3 (15%)	12 (10%)	0.52
Coronary artery disease, n (%)	11 (55%)	77 (65%)	0.41
Hypertension, n (%)	10 (50%)	78 (88%)	0.18
Hyperlipidaemia, n (%)	10 (50%)	65 (66%)	0.70
Periphery artery disease, n (%)	4 (20%)	26 (22%)	0.86
Diabetes mellitus, n (%)	2 (10%)	21 (18%)	0.40
Heart failure, n (%)	6 (30%)	20 (17%)	0.16
LAEDD (cm; mean ± SD)	4.66 ± 0.27	4.38 ± 0.38	**0.001** *

SD—standard deviation; AF–atrial fibrillation; LAD–left atrial diverticulum; LSSP–left-sided septal pouch; MRPV–middle right pulmonary vein; LAEDD, left atrial end diastolic diameter;

*—included in bivariate logistic regression analysis

**Table 2:** Binary logistic regression to determine independent predictors of AF recurrence.

Risk factor	Standard error	P-value	Hazard ratio	95% CI
Persistent AF subtype	0.616	0.010	4.8	1.5–16.3
LAD	0.584	0.043	2.7	1.0–8.4
Hypertension	0.540	0.122	2.3	0.8–6.6
Heart failure	0.609	0.060	3.1	1.0–10.4
LAEDD > 4,6 cm	0.597	0.062	3.1	0.9–9.8

AF–atrial fibrillation; LAD–left atrial diverticulum; LAEDD–left atrial end diastolic diameter; CI–confidence interval.

## 4. Discussion

The aim of this study was to investigate the impact of left atrial diverticula, left sided septal pouches and middle right pulmonary veins on recurrent atrial fibrillation after pulmonary vein isolation. First, the results demonstrated that LAD, LSSP and MRPV were common findings in cardiac CT scans with prevalence rates ranging between 11% and 29%. Second, our data suggest that LADs are independently associated with rAF. Third, persistent AF was a further independent risk factor for rAF.

LADs represent a common finding in patients with AF with an imaging prevalence of 29% in the current studies, which is within the range of previous studies [19, 20]. LADs can be found in any location of the left atrium, yet, our data indicate a preference for the right anterosuperior wall (Fig 2), which is in line with other recently published studies [20, 22, 31]. In accordance with Abbara et al., we did not find other cardiac abnormalities in patients with LADs; furthermore, there were no patients with multiple LADs [22]. There are a few case reports describing that LADs and other accessory appendages are a potential source of thromboembolism and a potential cause of perforation risk during ablation [19, 20, 32]. Interestingly, histopathological analyses could demonstrate that LADs contain trabeculated myocardium which raises the hypothesis that LAD may be contractile and a source of ectopic activity, which was reported in a documented case report by Kileen et al. [24]. However, the role of LADs in the pathomechanism of AF remains unclear. In the present study, there were no procedural complications during catheter ablation in all patients with LAD. A recent study with comparable design by De Ponti et. al. could not establish a significant correlation between LAD and AF or rAF. However, a differentiation between “true” diverticula, aneurysms and accessory appendages has not been done and findings were all subsumed as LAD. This causes a blurring regarding the meaningfulness of the clinical impact of “true” LAD, which were targeted in our study. In contrast to their described study, we used ECG-gated CT protocols and breathing commands to eliminate disruptive factors of image quality such as respiratory movements and pulsation artifacts of the heart. Another recently published study by Demir et al. could not prove a correlation between the presence of a LAD and rAF. The main difference to our study is that two different types of ablation procedures were used (cryoballoon- and radiofrequency ablation) and the amount of rAF was significantly higher in the group that underwent radiofrequency ablation. Therefore we only included patients that underwent laser pulmonary vein isolation to avoid this possible bias [33]. However, none of these studies could prove LAD an independent risk factor for rAF.

Since it is known that muscular sleeves from the left atrium can extend into the wall of pulmonary veins, PVI has been successfully used to disconnect the electrical connection between PVs and the left atrium [34–37]. We detected a total number of 15 MRPVs in our study cohort. However, in this study MRPV showed no statistical correlation to rAF. This is in concordance with the results reported by Khoueiry et al. who did not find a difference in the incidence of AF recurrence in patients undergoing cryoballon ablation (17%) or radiofrequency ablation (14,1%). Neither a significant impact of MRPV on procedural success could be detected [38].

To our knowledge this is the first study to investigate a possible correlation between LSSP and rAF after PVI. LSSP are defined as “kangaroo pouch-like structures”, that occur when the PFO is absent but the septum primum and septum secundum are not completely fused. A recently published study by Holda et al. could demonstrate that there is an association between the presence of LSSP and cryptogenic stroke. LSSP are also described as a trigger for AF [26]. This matches to other research projects regarding LSSP and AF, since several studies before and around the turn of the millennium have shown a correlation between redundant LSSP and AF, in fetuses [39–41]. Whether there is a causality between LSSP in adult patients and AF has not been finally clarified, at least our quite small data showed no correlation with AF recurrence after PVI.

The drawbacks of our study are the retrospective study design and the small study population number. Furthermore, every patient included in our study had a history of AF. A control group of healthy normal rhythmic patients carrying LAD’s without AF would be helpful to better understand clinical implications from our findings. Furthermore, it is important to emphasize that all patients in this study received antiarrhythmic drugs for 12 months after ablation. This interval seems quite long, but does not distort the long-term results, since several studies could proof, that AADs can prevent early atrial arrhythmias within 2 months after catheter ablation, but do not prevent late arrhythmias [42]. Another limitation is that in addition to LAD, there are also other outpouchings in the left atrium that could have been falsely characterized as LADs. These outpouchings are mainly described as left atrial accessory appendages (LAAAs). However, LAAAs are typically characterized by an irregular contour [22]. To minimize this potential bias, we only include LADs with a smooth contour. Despite these limitations this study describes post-procedural data with a follow up to 1 year.

In conclusion LAD, LSSP and MRPV are common findings during cardiac computed tomography of the heart. The role of LAD in rAF is still controversial, however logistic regression analysis revealed that presence of a LAD and persistent AF as potential independent risk factors for rAF in our study cohort. At this point, further prospective multicenter studies are needed to fully clarify the clinical significance of these structures within the left atrium and recurrence of atrial fibrillation.

## Supporting information

S1 Data (XLSX)

## Abbreviations

AF: atrial fibrillation
aPV: accessory pulmonary vein
CI: confidence interval
CMAP: compound motor action potential method
kV: kilovolt
LAD: left atrial diverticula
LAEDD: left atrial end diastolic diameter
LSSP: left sided septal pouch
mAs: milliampere-seconds
MDCT: multi detector computed tomography
ms: milliseconds
PACS: picture archiving and communication system
PFO: patent foramen ovale
PVI: pulmonary vein isolation procedure
SD: standard deviation
